# Sepsis Endotypes Defined by Lymphocyte Thresholds and Inflammation Inform Precision Immunomodulation

**DOI:** 10.1002/mco2.70561

**Published:** 2026-01-04

**Authors:** Zhongyi Sun, Li Li, Wenkang Gao, Han Gao, Liangyu Guo, Zhiyong Peng

**Affiliations:** ^1^ Department of Critical Care Medicine Zhongnan Hospital of Wuhan University Wuhan China; ^2^ Department of Pulmonary Medicine Zhongnan Hospital of Wuhan University Wuhan China; ^3^ Department of Orthopaedics Renmin Hospital of Wuhan University Wuhan China

**Keywords:** biomarker‐guided therapy, corticosteroids, immunomodulation, lymphocyte dysfunction, sepsis endotypes

## Abstract

Immunomodulatory therapies demonstrate variable efficacy in sepsis, suggesting biological heterogeneity inadequately captured by current stratification approaches. Although lymphopenia predicts mortality, functional thresholds and their interaction with inflammation remain poorly characterized. We investigated whether integrating lymphocyte status with systemic inflammation defines sepsis endotypes with differential treatment responsiveness. We retrospectively profiled 714 patients within 24 h using lymphocyte subsets and inflammatory biomarkers. Restricted cubic spline analysis revealed nonlinear associations between lymphocyte counts and mortality (*p* < 0.01), with steep risk increases at lower counts. Risk optimization identified critical thresholds at 374 cells/µL (total T cells), 340 cells/µL (CD4⁺), and 157 cells/µL (CD8⁺). Principal component analysis of inflammatory markers combined with lymphocyte stratification classified patients into four discrete endotypes with markedly divergent 28‐day survival (55%–58% vs. 82–87%, *p* < 0.001). Patients with immunosuppressed/hypo‐inflammatory endotype had higher survival among those who received corticosteroids (CD4⁺‐depleted: 84.4% vs. 75.6%, *p* < 0.001; T‐cell‐depleted: 78.7% vs. 72.3%, *p* = 0.006), whereas hyperinflammatory endotypes showed no such association. Integration of publicly available single‐cell (GSE167363) and bulk transcriptomics (GSE65682) datasets yielded a 15‐gene T‐cell dysfunction signature with external validation (CNP0004962, area under the curve [AUC] 0.76–0.85). These observational findings suggest that immune‐inflammatory co‐profiling identifies biologically distinct sepsis subgroups with differential treatment associations, generating testable hypotheses for prospective validation through endotype‐guided trials.

## Graphical abstract

Sepsis outcomes are shaped by immune–inflammatory imbalance. Integrative profiling identified critical lymphocyte thresholds and defined endotypes linking immune depletion with inflammation. Patients with low inflammation but lymphocyte loss benefited from corticosteroids, while hyperinflammatory endotypes did not. These findings establish a clinically actionable framework for precision immunomodulation, bridging immune phenotyping with individualized sepsis therapy.

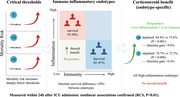

## 1 Introduction

Sepsis affects more than 49 million people and causes approximately 11 million deaths annually worldwide [1]. Despite more than a hundred clinical trials evaluating immunomodulatory interventions, few have shown consistent benefit when applied uniformly to unselected populations [2, 3]. This failure reflects not inadequate pharmacology agents such as corticosteroids and interleukin‐7 (IL‐7) possess demonstrable biological activity but rather the profound heterogeneity of host immune responses [4, 5]. Sepsis triggers divergent immune trajectories: hyperinflammation characterized by excessive cytokine production and tissue injury in some patients, and immunosuppression marked by lymphocyte depletion and secondary infections in others [6, 7]. These opposing states impose different therapeutic requirements; administering immunosuppressive therapy to immunocompromised patients, or immunostimulatory agents to hyperinflammatory patients, may confer minimal benefit or cause harm [8, 9]. Precision immunomodulation requires identifying biologically distinct patient subgroups immune‐inflammatory phenotypes representing discrete endotypes that exhibit differential treatment responsiveness [10, 11, 12].

Recent trial evidence supports this paradigm. The Adjunctive Corticosteroid Treatment in Critically Ill Patients with Septic Shock (ADRENAL) found no mortality benefit with hydrocortisone in unselected patients [13], whereas the Activated Protein C and Corticosteroids for Human Septic Shock (APROCCHSS) trial demonstrated significant mortality reduction with combination corticosteroid therapy [14]. Post hoc analyses suggest potential explanations: the Vasopressin versus Norepinephrine as Initial Therapy in Septic Shock (VANISH) indicated potential benefits in subgroups with relative adrenal insufficiency [15], while the IL‐7 in Septic Shock (IRIS‐7) trial showed efficacy primarily in profoundly lymphopenic patients [16]. These observations suggest therapeutic efficacy may depend on quantifiable biological states rather than sepsis diagnosis alone [15, 16]. However, retrospective subgroup identification through post hoc analyses differs fundamentally from prospective patient classification using predefined, readily available measurements such as lymphocyte subset counts and inflammatory markers [17, 18]. How cellular immune deficits interact with systemic inflammatory burden to identify treatment‐responsive subgroups remains incompletely characterized.

Lymphocyte status and systemic inflammation represent potentially independent dimensions of sepsis pathophysiology. Lymphopenia associates with increased mortality [19, 20], reflecting apoptosis, functional exhaustion, and impaired regeneration [21, 22]. However, patients with comparable lymphocyte depletion may exhibit markedly different inflammatory profiles ranging from minimal cytokine production to robust inflammatory activity [23, 24]. These patterns may reflect distinct biological states with different therapeutic requirements. Patients presenting with lymphopenia and low systemic inflammation might represent an immunosuppressed phenotype that could benefit from immunostimulation, whereas those with lymphopenia alongside persistent hyperinflammation may require alternative approaches [23, 24, 25]. Integrating cellular immune status with inflammatory activity rather than evaluating either dimension in isolation may identify discrete endotypes with distinct treatment needs.

Several considerations extend this framework. Different lymphocyte subsets‐CD4^+^T cells, CD8^+^ T cells, natural killer cells, and B cells contribute distinct mechanisms to antimicrobial defense [26, 27], and their depletion patterns may provide more granular endotypic information than total lymphocyte counts alone. Contemporary molecular profiling offers additional depth: single‐cell transcriptomics identify dysfunction states within T‐cell populations [28, 29], while bulk transcriptomics generate gene expression signatures suitable for clinical deployment [30, 31]. Whether systematic integration of cellular immune profiling, inflammatory biomarkers, and molecular signatures can prospectively identify patients most likely to benefit from specific immunomodulatory interventions remains unestablished.

## 2 Results

### 2.1 Profound Lymphopenia Distinguishes Non‐Survivors From Survivors in Sepsis

Among 714 sepsis patients enrolled, 186 (26.1%) died within 28 days of ICU admission (Table 1). Non‐survivors were older (median 60.0 vs. 57.0 years, *p* = 0.007) and more frequently male (75.1% vs. 67.1%, *p* = 0.037). They exhibited significantly higher illness severity scores (APACHE II: 26 vs. 20, *p* < 0.001; SOFA: 11.0 vs. 7.5, *p* < 0.001). Non‐survivors demonstrated markedly lower lymphocyte counts across all subsets: total T cells (258.0 vs. 437.0/µL, *p* < 0.001), CD4^+^ T cells (118.5 vs. 190.0/µL, *p* < 0.001), CD8^+^ T cells (110.6 vs. 179.0/µL, *p* < 0.001), B cells (54.0 vs. 75.0/µL, *p* = 0.024), and NK cells (42.2 vs. 71.0/µL, *p* = 0.003). Non‐survivors also exhibited lower lymphocyte percentages among total white blood cells (2.4% vs. 3.9%, *p* = 0.001), underscoring the profound lymphopenia characterizing fatal sepsis outcomes.

**TABLE 1:** Baseline characteristics of sepsis patients stratified by 28‐day survival status.

Variable	Total (*n* = 714)	Survival (*n* = 528)	Non‐survival (*n* = 186)	*p* value
**Demographics**				
Age, years, median (IQR)	58.0 (45.0, 69.0)	57.0 (41.8, 68.0)	60.0 (50.0, 69.8)	0.007
Male sex, *n* (%)	494 (69.2)	354 (67.1)	140(75.1)	0.037
**Vital signs**				
Heart rate, bpm, median (IQR)	160.0 (137.0, 192.0)	157.5 (134.8, 191.0)	168.0 (143.3, 200.0)	0.002
SBP, mmHg, median (IQR)	79.5 (67.0, 92.0)	81.0 (69.0, 93.0)	74.0 (60.0, 90.8)	0.001
DBP, mmHg, median (IQR)	48.0 (38.0, 58.0)	49.0 (41.0, 58.0)	45.5 (32.0, 57.0)	0.008
MAP, mmHg, median (IQR)	59.0 (46.0, 68.0)	59.0 (47.0, 68.0)	56.0 (41.5, 68.0)	0.040
**Lymphocyte subsets**				
T cells, %, median (IQR)	68.0 (56.5, 77.5)	68.5 (57.8, 78.4)	66.2 (52.7, 75.3)	0.003
CD4⁺ T cells, %, median (IQR)	30.5 (17.0, 42.3)	30.7 (17.4, 41.8)	28.7 (16.6, 43.3)	0.809
CD8⁺ T cells, %, median (IQR)	27.5 (18.4, 45.3)	28.5 (18.7, 47.3)	25.2 (16.5, 39.6)	0.014
B cells, %, median (IQR)	12.6 (5.2, 23.2)	12.3 (5.2, 22.4)	12.9 (5.2, 24.1)	0.663
NK cells, %, median (IQR)	11.1 (5.9, 19.0)	11.5 (6.3, 18.5)	9.7 (5.1, 19.8)	0.275
T cells, cells/µL, median (IQR)	374.0 (200.5, 698.5)	437.0 (221.0, 805.0)	258.0 (143.7, 443.3)	< 0.001
CD4⁺ T cells, cells/µL, median (IQR)	170.0 (64.0, 340.0)	190.0 (71.0, 390.0)	118.5 (53.0, 251.8)	< 0.001
CD8⁺ T cells, cells/µL, median (IQR)	157.0 (77.5, 323.0)	179.0 (91.0, 361.0)	110.6 (53.5, 190.0)	< 0.001
B cells, cells/µL, median (IQR)	69.0 (24.0, 156.5)	75.0 (26.0, 169.0)	54.0 (20.3, 139.8)	0.018
NK cells, cells/µL, median (IQR)	64.0 (25.5, 141.0)	71.0 (30.0, 155.0)	42.2 (17.0, 102.0)	< 0.001
**Severity scores**				
APACHE II, median (IQR)	21.00 (15.0, 28.0)	20.0 (15.0, 25.0)	26.0 (20.0, 31.0)	< 0.001
SOFA, median (IQR)	8.0 (5.0, 12.0)	7.5 (5.0, 11.0)	11.00 (8.00, 15.0)	< 0.001
**Comorbidities**				
COPD, *n* (%)	15 (2.1)	12 (2.3)	3 (1.6)	0.808
Hypertension, *n* (%)	130 (18.2)	97 (18.4)	33 (17.7)	0.851
Heart failure, *n* (%)	242 (33.9)	160 (30.3)	82 (44.1)	< 0.001
Liver disease, *n* (%)	298 (41.7)	220 (41.7)	78 (41.9)	0.949
Renal failure, *n* (%)	327 (45.8)	234 (44.3)	93 (50)	0.211
Cerebrovascular disease, *n* (%)	135 (18.9)	93 (17.6)	42 (22.6)	0.168
Diabetes, *n* (%)	115 (16.1)	83 (15.7)	32 (17.2)	0.721
Cancer, *n* (%)	157 (22.0)	110 (20.8)	47 (25.3)	0.249
**Complete blood count**				
WBC, ×10^3^/µL, median (IQR)	22.75 (11.1, 68.9)	21.4 (10.2, 66.7)	24.3 (12.6, 72.3)	0.102
Platelets, ×10^3^/µL, median (IQR)	63.5 (25.0, 145.5)	73.0 (32.0, 160.8)	31.5 (15.0, 80.0)	< 0.001
Neutrophil, %, median (IQR)	92.0 (85.2, 95.4)	91.2 (84.0, 94.8)	94.4 (89.4, 96.4)	< 0.001
Neutrophil, ×10^3^/µL, median (IQR)	12.1 (6.9, 19.5)	11.7 (6.6, 19.0)	13.8 (8.0, 21.4)	0.024
Lymphocyte, %, median (IQR)	3.6 (1.9, 7.7)	3.9 (2.3, 8.0)	2.4 (1.3, 5.2)	< 0.001
Lymphocyte, ×10^3^/µL, median (IQR)	0.3 (0.2, 0.5)	0.3 (0.2, 0.6)	0.2 (0.1, 0.4)	< 0.001
**Organ function and metabolic**				
Creatinine, µmol/L, median (IQR)	106.9 (71.0, 257.7)	94.0 (67.6, 226.0)	157.2 (86.6, 326.2)	< 0.001
Total bilirubin, µmol/L, median (IQR)	24.0 (13.5, 57.3)	20.2 (12.6, 44.4)	38.7 (20.7, 119.9)	< 0.001
AST/ALT ratio, median (IQR)	2.3 (1.5, 3.8)	2.1 (1.4, 3.4)	3.0 (1.8, 5.0)	< 0.001
Albumin, g/L, median (IQR)	34.3 (30.6, 38.4)	34.5 (30.7, 38.3)	33.9 (29.8, 38.5)	0.315
Calcium, mmol/L, median (IQR)	2.2 (2.1, 2.4)	2.2 (2.1, 2.4)	2.3 (2.1, 2.5)	0.035
Magnesium, mmol/L, median (IQR)	1.0 (0.9, 1.1)	1.0 (0.9, 1.1)	1.1 (0.9, 1.2)	< 0.001
Sodium, mmol/L, median (IQR)	142.3 (138.9, 149.0)	141.6 (138.5, 147.0)	147.1 (140.3, 152.3)	< 0.001
Potassium, mmol/L, median (IQR)	4.6 (4.2, 5.2)	4.5 (4.2, 5.0)	4.9 (4.4, 5.5)	< 0.001
**Coagulation**				
Fibrinogen, mg/mL, median (IQR)	385.5 (148.0, 483.0)	391.0 (165.8, 491.0)	288.0 (108.5, 451.3)	< 0.001
PT, s, median (IQR)	17.1 (15.0, 21.3)	16.6 (14.9, 19.7)	19.0 (15.6, 27.7)	< 0.001
D‐dimer, ng/mL, median (IQR)	3054.5 (1122.0, 6913.3)	2795.5 (945.5, 5806.5)	4155.5 (2262.5, 10,932.8)	< 0.001
APTT, s, median (IQR)	36.5 (31.8, 48.0)	35.1 (31.5, 43.6)	45.4 (35.1, 77.3)	< 0.001
**Inflammation and acid–base**				
CRP, mg/L, median (IQR)	99.4 (38.6, 179.2)	92.55 (34.4, 173.5)	116.3 (45.0, 203.1)	0.029
PCT, ng/mL, median (IQR)	7.7 (1.8, 37.5)	6.3 (1.4, 28.5)	17.7 (4.8, 58.6)	< 0.001
Lactate, mmol/L, median (IQR)	1.8 (0.9, 3.7)	1.7 (0.9, 3.4)	2.3 (1.1, 5.4)	0.001
IL‐6, pg/mL, median (IQR)	216.0 (72.7, 1348.3)	159.3 (58.9, 868.0)	827.9 (142.8, 5000.0)	< 0.001

*Note*: Data are presented as median (interquartile range) or *n* (%).

Abbreviations: ALT, alanine aminotransferase; APACHE, Acute Physiology and Chronic Health Evaluation; APTT, activated partial thromboplastin time; AST, aspartate aminotransferase; COPD, chronic obstructive pulmonary disease; CRP, C‐reactive protein; DBP, diastolic blood pressure; IL‐6, interleukin‐6; IQR, interquartile range; MAP, mean arterial pressure; NK, natural killer; PCT, procalcitonin; PT, prothrombin time; SBP, systolic blood pressure; SOFA, Sequential Organ Failure Assessment; WBC, white blood cell.

### 2.2 Higher Lymphocyte Counts Confer Dose‐Dependent Survival Advantage

**FIGURE 1:**
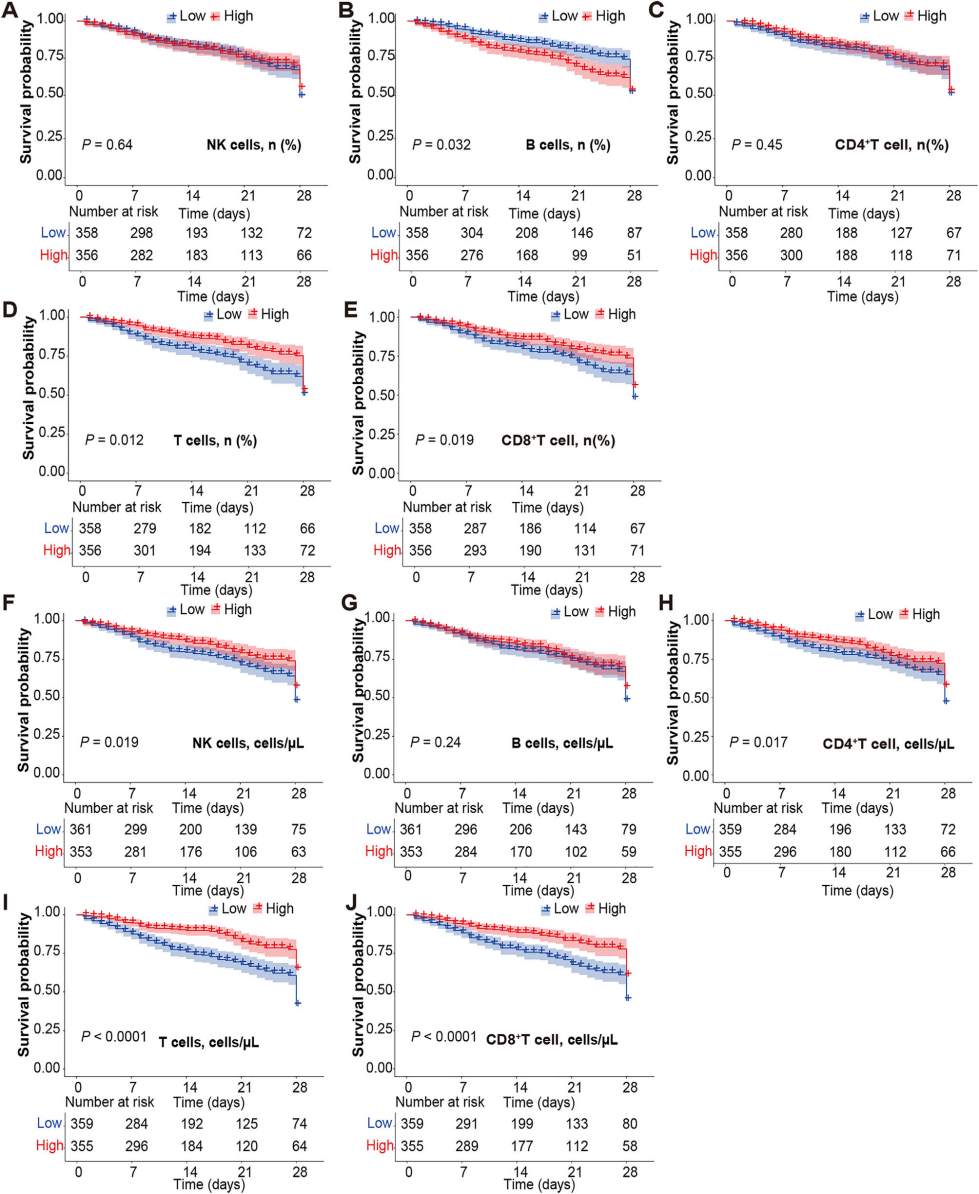
Kaplan–Meier survival by baseline lymphocyte subsets. (A–E) Percentages of NK cells, B cells, CD4⁺ T cells, total T cells, and CD8⁺ T cells stratified by cohort median (high ≥ median; low < median). (F–J) Absolute counts for the same subsets (cells/µL), stratified by cohort median. Curves show 28‐day survival; tick marks denote censoring; shaded bands indicate 95% CIs. *p* values are from two‐sided log‐rank tests (notably T‐cell% *p* = 0.012 and CD8⁺ T% *p* = 0.019).

Consistent with these patterns, Kaplan–Meier survival analyses demonstrated that higher lymphocyte counts were associated with significantly improved survival. Patients in the higher strata of total T‐cell count, CD4⁺ T‐cell count, or CD8⁺ T‐cell counts exhibited markedly superior 28‐day survival compared with those having low counts (log‐rank *p* < 0.05 for each, Figure 1A–J). Similarly, patients with higher T‐cell and CD8^+^ T‐cell percentages (relative to total lymphocytes) demonstrated superior survival probabilities (log‐rank *p* = 0.012 and *p* = 0.019, respectively).

### 2.3 T‐Cell Count–Mortality Relationship Exhibits Critical Threshold Effects

**FIGURE 2:**
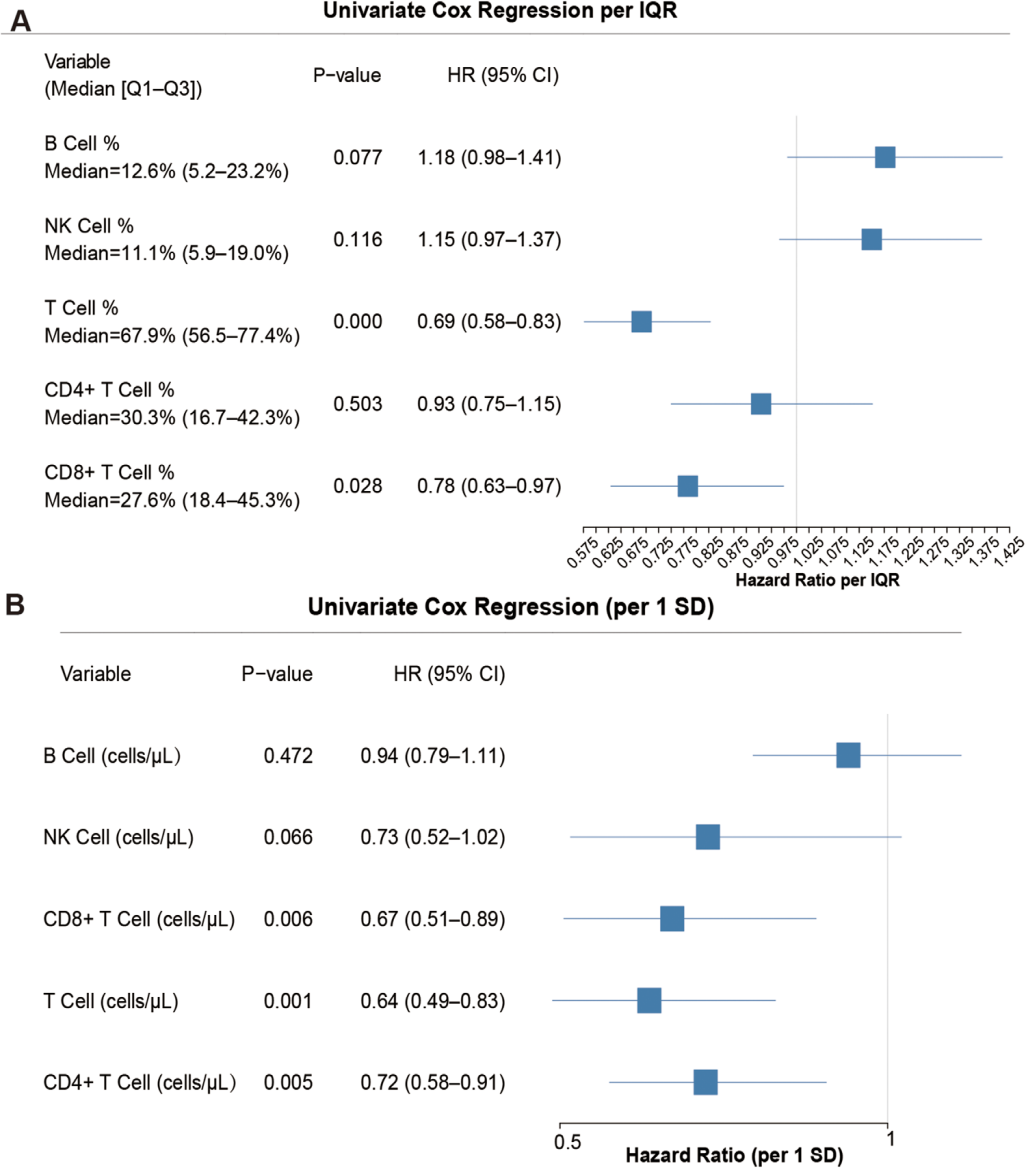
Univariate Cox regression analysis examining the associations between lymphocyte subset distributions and all‐cause mortality. (A) Forest plot of univariate Cox regression for percentage‐based lymphocyte subset metrics. HR per interquartile range (IQR) increase is shown with 95% CI. Variables include B cell %, NK cell %, T cell %, CD4^+^ T cell %, and CD8^+^ T cell % (median values and IQR shown in the table). The vertical dashed line at HR = 1.0 indicates no association with mortality. HR values < 1.0 indicate protective associations; values > 1.0 indicate increased mortality risk. *p*‐values were calculated using univariate Cox proportional hazards regression. (B) Forest plot of univariate Cox regression for absolute count‐based lymphocyte subset metrics. HR per one standard deviation (SD) increase in absolute cell counts (cells/µL) are shown with 95% CIs for B cells, NK cells, CD8^+^ T cells, T cells, and CD4^+^ T cells. The vertical dashed line at HR = 1.0 represents no association. *p* values and HR were derived from univariate Cox proportional hazards models.

**FIGURE 3:**
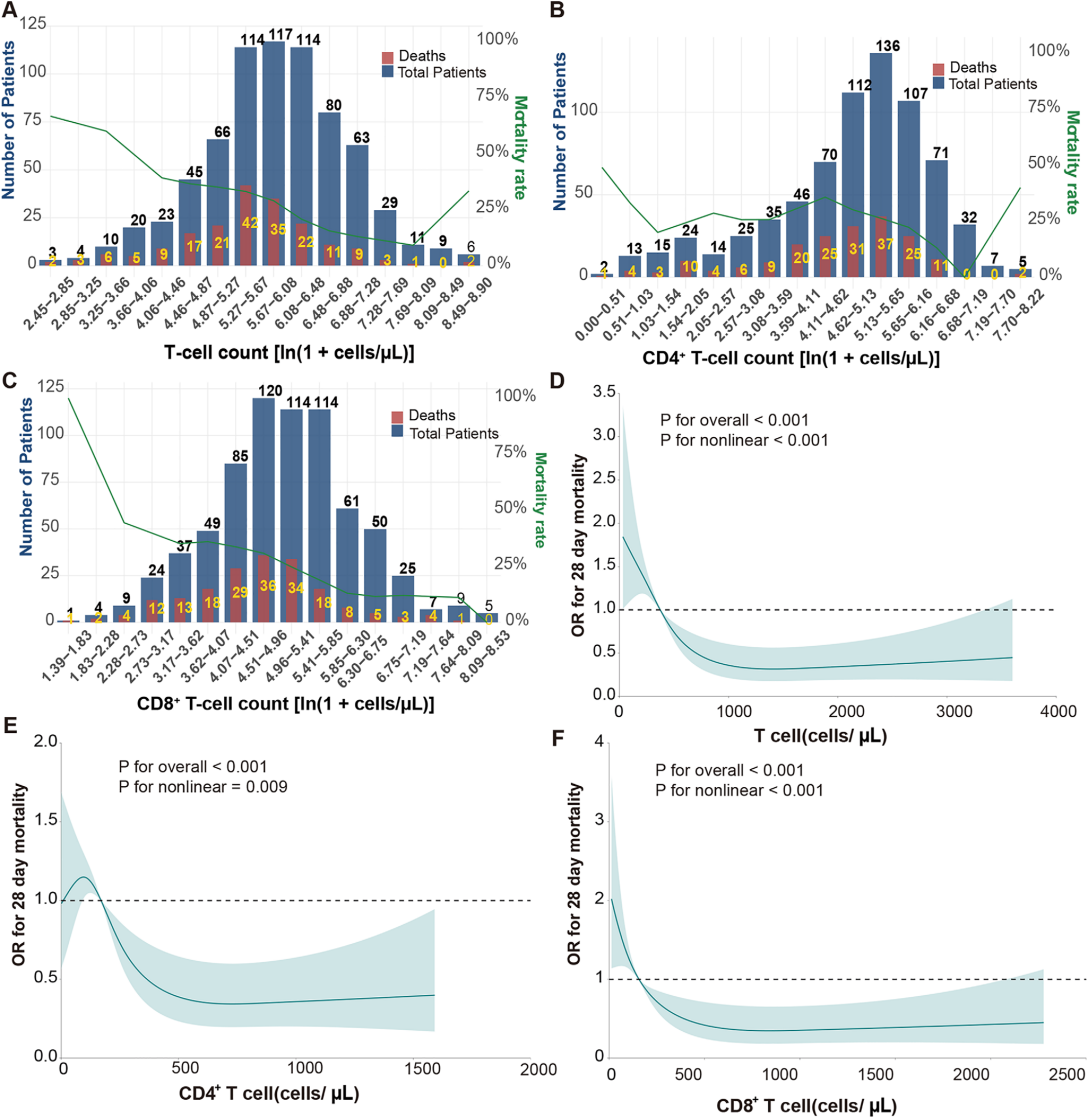
Nonlinear dose–response relationships between lymphocyte subset counts and 28‐day mortality. (A–C) Distribution of patients and mortality across transformed lymphocyte count intervals. For each lymphocyte subset. (A) Total T cells, (B) CD4⁺ T cells, and (C) CD8⁺ T cells stacked bars show the number of patients (blue) and deaths (red) within equal‐width intervals of ln (1 + cells/µL). Green lines indicate LOWESS‐smoothed estimates of 28‐day mortality within each interval. Numeric labels above the bars denote the total number of patients in that interval; red labels within bars denote the number of deaths. (D–F) Restricted cubic spline curves showing associations between absolute lymphocyte counts and 28‐day mortality. ORs for 28‐day mortality are plotted as a function of absolute cell counts (cells/µL) for (D) total T cells, (E) CD4^+^ T cells, and (F) CD8^+^ T cells. The horizontal dashed line at OR = 1.0 represents the reference level; shaded areas represent 95% CI. OR and 95% CI were estimated from logistic regression models with restricted cubic splines, fitted with four knots at the fifth, 35th, 65th, and 95th percentiles. *p* values for overall association and nonlinearity are shown in each panel.

In univariate Cox regression, higher lymphocyte counts conferred significant mortality protection. Each standard deviation increases in total T‐cell count yielded a hazard ratio (HR) of 0.64 (95% CI: 0.49–0.85, *p* = 0.001), indicating a 36% relative risk reduction. Similar protective associations emerged for CD4⁺ T cells (HR 0.72, 95% CI: 0.58–0.91, *p* = 0.005) and CD8⁺ T cells (HR 0.67, 95% CI: 0.51–0.89 *p* = 0.006). Higher T‐cell and CD8⁺ T‐cell percentages are likewise associated with reduced mortality risk (Figure 2A,B). Importantly, integrated visualization using locally weighted scatterplot smoothing (LOWESS), distribution histograms, and restricted cubic spline (RCS) modeling demonstrated a significant nonlinear association between lymphocyte counts and mortality. As shown in Figure 3A–C, mortality (LOWESS‐smoothed green curves) declined sharply with increasing lymphocyte counts from the lowest ranges, with the highest mortality (>50%) observed in patients with severe lymphocyte depletion. Most patients were concentrated within intermediate ranges of the transformed counts [ln (1 + cells/µL): total T cells 4.87–6.48, CD4⁺ T cells 3.59–5.65, and CD8⁺ T cells 4.07‐5.85], within which the LOWESS curves indicated substantially lower mortality (approximately 0%–25%).

RCS modeling (Figure 3D–F) quantified these nonlinear associations, revealing characteristic L‐shaped dose–response curves. For total T cells, mortality risk was markedly elevated at very low counts (<500 cells/µL), declined steeply between 500 and 1000 cells/µL, and plateaued at counts exceeding 1000 cells/µL (Figure 3D). Similar patterns emerged for CD4^+^ and CD8^+^ T cells (Figure 3E,F). Formal statistical testing confirmed significant departures from linearity for all three subsets (*p* for nonlinear < 0.001 for total T cells and CD8⁺ T cells, *p* = 0.009 for CD4⁺ T cells; *p* for overall association < 0.001 for all), consistent with critical threshold effects below which mortality risk increases exponentially.

### 2.4 Actionable Lymphocyte Thresholds Define High‐Risk Sepsis Subgroups

Risk optimization methods identified clear lymphocyte count thresholds that stratified patients into high‐ and low‐risk groups (Table 2). A total T‐cell count below 374 cells/µL was associated with significantly elevated mortality risk (odds ratio [OR]: 2.84, 95% CI: 1.99–4.04, *p* < 0.001 for patients below vs. above this threshold). Similarly, a CD4⁺ T‐cell count below 340 cells/µL (OR: 2.28, 95% CI: 1.46–3.55, *p* < 0.001) and a CD8⁺ T‐cell count below 157 cells/µL (OR: 2.66, 95% CI: 1.87‐3.78, *p* < 0.001) defined high‐risk strata. These represent actionable immune thresholds where mortality risk increases sharply.

**TABLE 2:** Clinical thresholds for T lymphocyte risk stratification.

T cell subset	Optimal threshold (cells/µL)	Below threshold (deaths/total)	Above threshold (deaths/total)	OR (95% CI)	*χ* ^2^	*p* value
CD8^+^ T cells	157	126/358	60/356	2.66 (1.87–3.78)	30.95	< 0.001
Total T cells	374	128/358	58/356	2.84 (1.99–4.04)	34.85	< 0.001
CD4^+^ T cells	340	159/536	27/178	2.28 (1.46–3.55)	13.74	< 0.001

*Note*: Thresholds optimized for maximum clinical discrimination.

Abbreviations: CI, confidence interval; OR, odds ratio.

### 2.5 Immune–Inflammatory Endotypes Identify Subgroups With Divergent Survival

**FIGURE 4:**
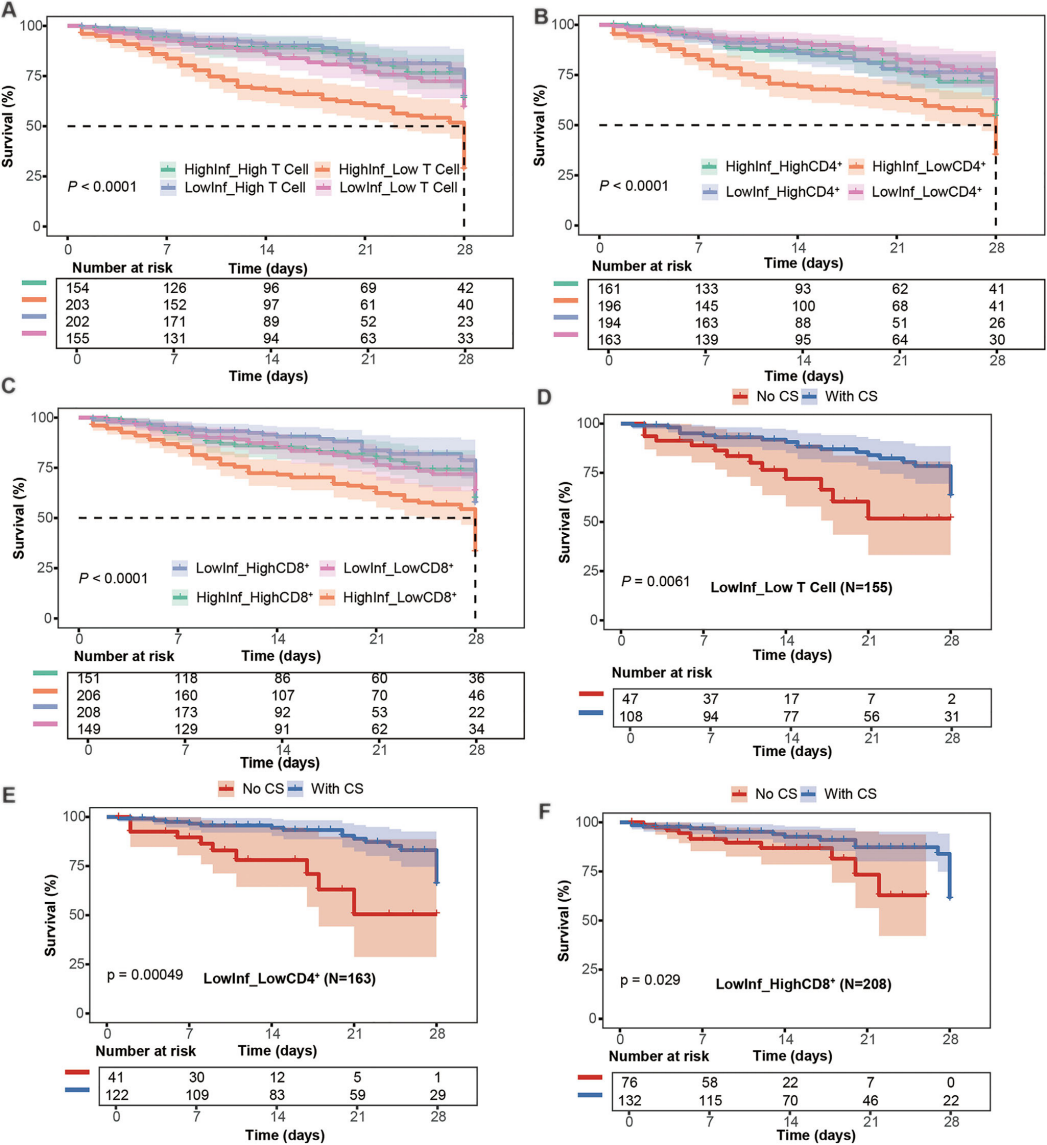
Immune‐inflammatory endotypes stratify 28‐day survival and corticosteroid response. (A–C) Kaplan–Meier survival curves by immune‐inflammatory endotypes. Patients were classified by systemic inflammation status (high vs. low, dichotomized at the median of the composite index from principal component analysis of CRP, IL‐6, and PCT) and lymphocyte counts (high vs. low, dichotomized at the median). (A) Total T‐cell‐based endotypes. (B) CD4⁺ T‐cell‐based endotypes. (C) CD8⁺ T‐cell‐based endotypes. Shaded areas represent 95% confidence intervals. Numbers at risk are shown at 7‐day intervals. *p*‐values by log‐rank test. (D–F) Corticosteroid associations in propensity score‐matched cohorts within low‐inflammation endotypes. Blue curves: corticosteroid‐treated patients; red curves: no corticosteroids. Matching variables included baseline disease severity, organ support, infection characteristics, immune‐inflammatory endotype, and team. (D) Low inflammation + low T cells (*N* = 155). (E) Low inflammation + low CD4⁺ (*N* = 163). (F) Low inflammation + high CD8⁺ (*N* = 208). Shaded areas represent 95% CI. *p*‐values by log‐rank test.

Recognizing that lymphocyte depletion and systemic inflammation may represent partially independent pathophysiologic axes in sepsis, we investigated whether their integration could refine prognostic stratification. We constructed a composite inflammatory index using principal component analysis (PCA) of three established biomarkers C‐reactive protein (CRP), interleukin‐6 (IL‐6), and procalcitonin (PCT), with the first component capturing 73.2% of the variance. This continuous inflammatory score was dichotomized at the median to classify patients as having high versus low systemic inflammation. In parallel, absolute counts of each lymphocyte subset were dichotomized at their respective medians to define low versus high lymphocyte abundance. Cross‐classifying these two axes generated four discrete immune‐inflammatory endotypes for each T‐cell compartment. Kaplan–Meier analysis revealed that these endotypes exhibited profound and reproducible survival differences (all *p* < 0.001, Figure 4A–C). Patients presenting with the dual burden of high inflammation and lymphocyte depletion experienced uniformly poor outcomes: 28‐day survival was only 55.2% (112/203) when stratified by total T cells, 57.7% (113/196) by CD4⁺ counts, and 56.8% (117/206) by CD8⁺ counts. In stark contrast, patients with low inflammation and preserved lymphocyte counts achieved survival rates exceeding 82% across all compartments: 86.6% (175/202) for high total T cells, 82.2% (134/163) for high CD4⁺ cells, and 86.1% (179/208) for high CD8⁺ cells. The remarkable consistency of this pattern with survival consistently clustering around 55%–58% in high‐risk versus 82%–87% in low‐risk endotypes generated absolute survival differences exceeding 30 percentage points. These findings establish immune‐inflammatory co‐profiling as a robust framework for identifying biologically and prognostically distinct sepsis subgroups, laying the foundation for subsequent treatment response analysis.

### 2.6 Corticosteroid Benefit Restricted to Low‐Inflammation, Lymphopenic Endotypes

After propensity score matching (PSM) to balance baseline severity, organ support, infection source/pathogen, immune‐inflammatory endotype, and team, corticosteroid use was associated with higher 28‐day survival in endotypes characterized by low systemic inflammation and lymphocyte depletion (Figure 4D–F). In the low‐inflammation + low total T‐cell endotype, matched patients who received corticosteroids had a 28‐day survival of 78.7% compared with 72.3% in matched patients who did not receive corticosteroids (absolute benefit +6.4%, *p* = 0.006). A similar association was observed in the low‐inflammation + low CD4⁺ T‐cell endotype, where survival was 84.4% with corticosteroids versus 75.6% without (absolute difference 8.8%, *p* < 0.001). In the low‐inflammation + high CD8⁺ T‐cell endotype, where baseline survival was already high, the survival difference between steroid‐treated and non‐treated patients was small (86.4% vs. 85.5%, +0.9%, *p* = 0.029). Notably, no survival advantage from steroids was observed in any high‐inflammation endotype. These findings indicate that the observed survival differences linked to corticosteroid exposure were most evident in patients with low systemic inflammation and marked lymphocyte depletion; these results represent adjusted associations rather than proven causal effects.

### 2.7 Transcriptomic Profiling Reveals Sepsis‐Induced T‐Cell Exhaustion and Metabolic Dysfunction

**FIGURE 5:**
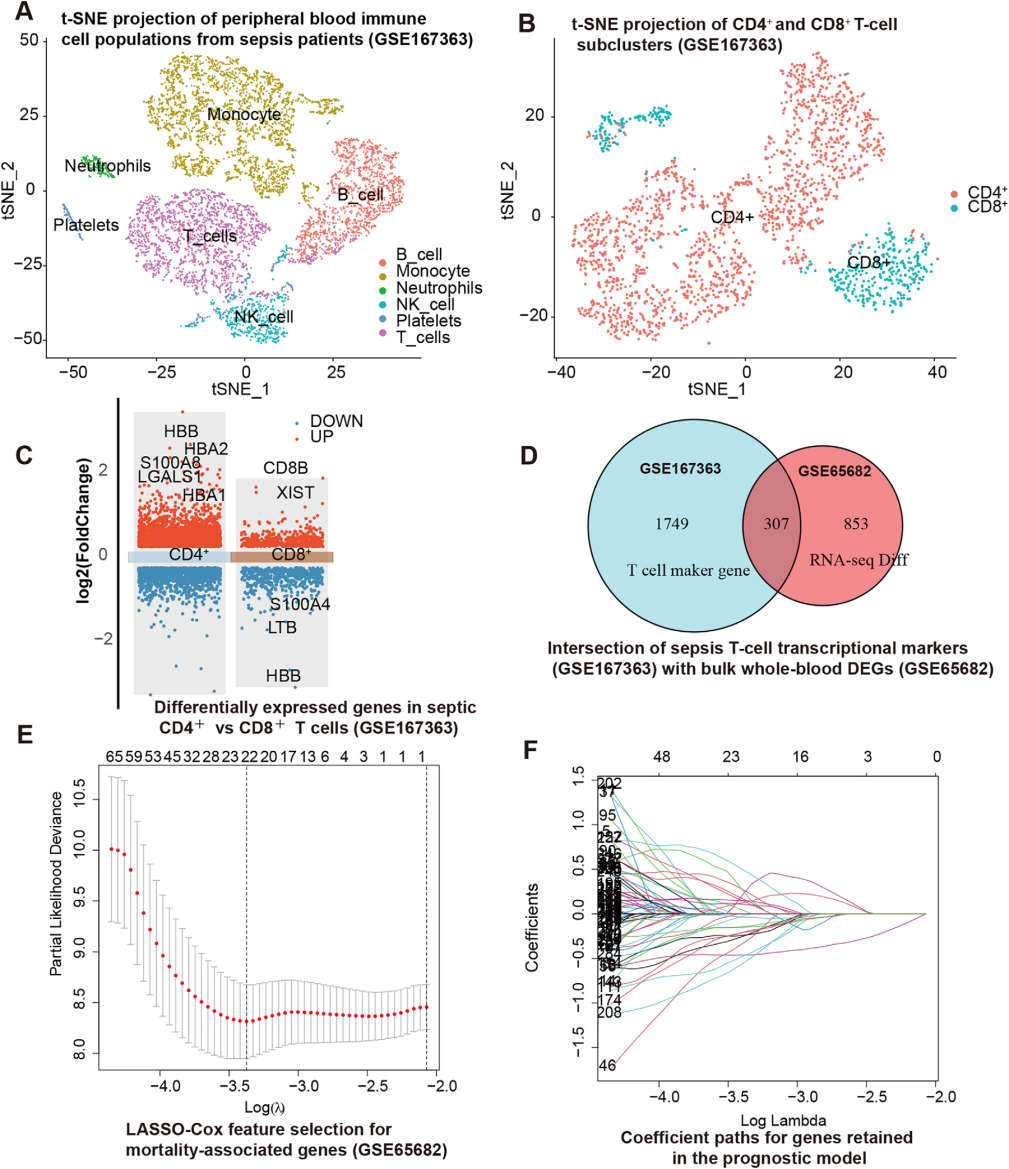
Integrative single‐cell and bulk transcriptomic analysis identifies a T‐cell‐based prognostic signature. (A) t‐SNE projection of peripheral blood immune cell populations from sepsis patients (GSE167363). Major immune cell types identified by unsupervised clustering are color‐coded: T cells (purple), B cells (red), NK cells (cyan), monocytes (yellow), neutrophils (green), and platelets (pink). Each dot represents a single cell. (B) t‐SNE projection of CD4⁺ (red) and CD8⁺ (cyan) T‐cell subclusters from sepsis patients (GSE167363). Each dot represents an individual T cell. (C) Volcano plot of differentially expressed genes in CD4⁺ versus CD8⁺ T cells. Log_2_ fold change is plotted on the x‐axis. Upregulated genes are shown in red, downregulated genes in blue. Key marker genes are labeled. (D) Venn diagram showing the intersection of T‐cell transcriptional markers (GSE167363) with bulk whole‐blood differentially expressed genes (GSE65682). The overlap of 307 genes was used for prognostic modeling. (E) LASSO‐Cox feature selection curve. Partial likelihood deviance (*y*‐axis) is plotted as a function of log(λ) (*x*‐axis) with 10‐fold cross‐validation. Numbers at the top indicate non‐zero coefficients. Error bars represent standard errors. Vertical dashed lines indicate optimal *λ* values. (F) LASSO coefficient paths for candidate genes. Each line represents a gene's coefficient trajectory as regularization increases. The 15 genes with non‐zero coefficients at the optimal *λ* (vertical dashed line) comprise the final prognostic signature. Differential expression in panel C was assessed as described in Section 4, and LASSO feature selection in panels E–F was performed in a Cox proportional hazards model with 10‐fold cross‐validation.

We performed single‐cell RNA sequencing (scRNA‐seq) of peripheral blood mononuclear cells (PBMCs) from five ICU patients with sepsis at admission (GSE167363). Quality control, integration, and cell‐type annotation are summarized in Figure S1. After filtering and integration, unsupervised clustering resolved 10 clusters that were grouped into six major immune populations—T cells, B cells, NK cells, monocytes, neutrophil‐like granulocytes, and platelets—based on canonical markers and SingleR (Figure S1C–F). t‐SNE projection (Figure 5A) similarly resolved these lineages, including T cells (*CD3D*⁺), B cells (*MS4A1*⁺), NK cells (*NKG7*⁺/*KLRD1*⁺), monocytes (*LYZ*⁺/*S100A8*⁺), neutrophil‐like granulocytic clusters (*S100A8*⁺/*S100A9*⁺), and platelet/megakaryocytic fragments (*PPBP*⁺). Donor‐level composition of these populations is shown in Figure S2A. Within the T‐cell compartment, CD4⁺ and CD8⁺ subsets were defined by canonical markers (*CD4*, *SELL*, *CCR7*, *ITK*, *IL7R*; *CD8A*, *CD8B*, *CD27*, *CRTAM*, and *GZMH*), as illustrated in the dot plots and feature plots in Figure S2B,C, which provided the basis for subsequent T‐cell‐focused analyses.

Because our clinical cohort analysis demonstrated that reduced circulating T‐cell counts were strongly associated with worse 28‐day survival, we next sought mechanistic insight into T‐cell dysfunction using this independent scRNA‐seq dataset. We focused on the adaptive immune compartment by subsetting *CD3D*⁺ T cells and performing higher resolution re‐clustering. This approach separated CD4⁺ and CD8⁺ T‐cell states based on established markers (*CD4*, *IL7R*, *CCR7* for CD4⁺ helper/central memory‐like populations; *CD8A*, *CD8B*, *GZMH* for cytotoxic CD8⁺ populations) (Figure 5B). Differential expression analysis between these septic T‐cell states revealed coordinated programs of exhaustion, impaired effector function, and metabolic stress (Figure 5C,D).

### 2.8 A 15‐Gene T‐Cell Dysfunction Signature Stratifies Sepsis Mortality Risk Across Cohorts

**FIGURE 6:**
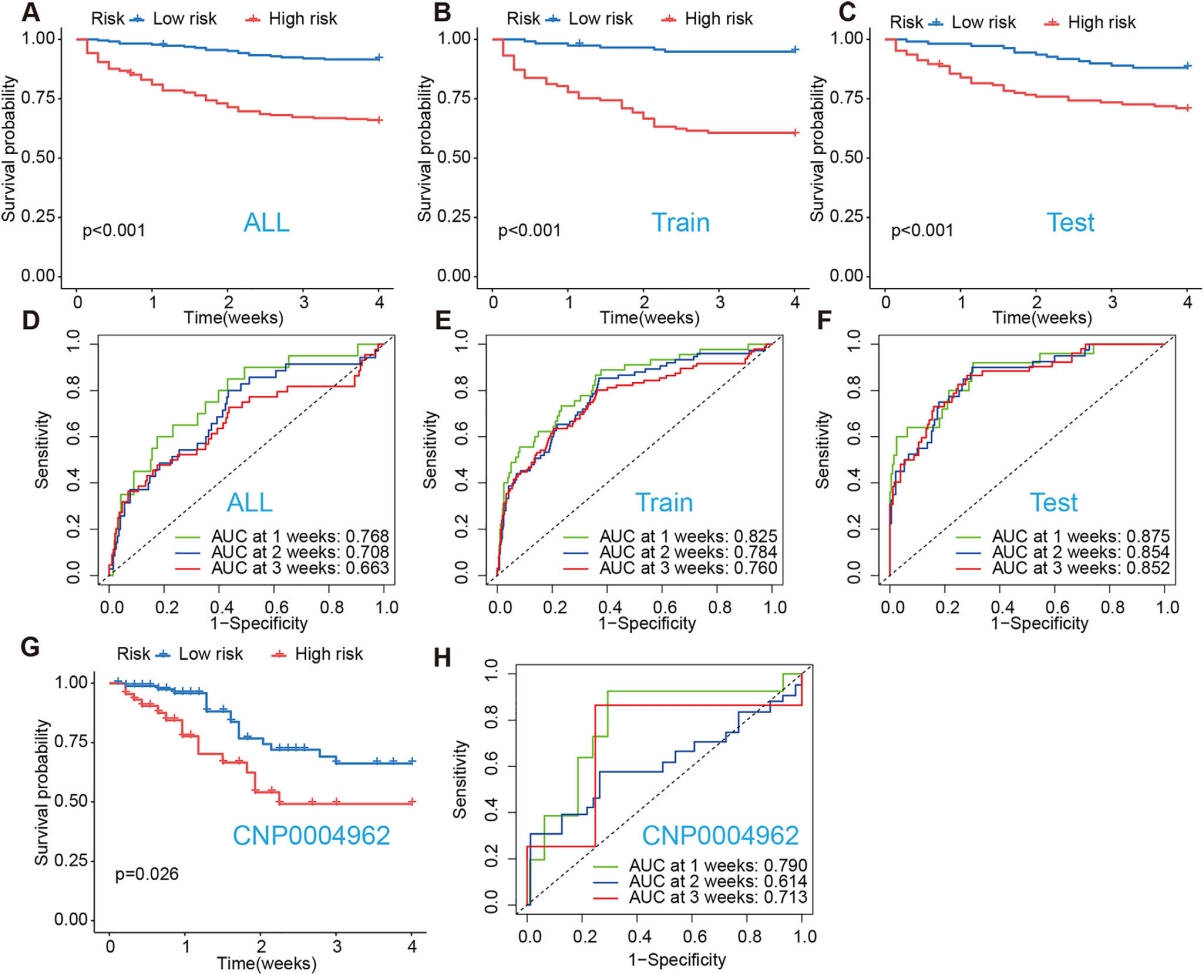
Validation of the 15‐gene prognostic signature in discovery and external cohorts. (A–C) Kaplan–Meier survival curves for high‐risk (red) and low‐risk (blue) groups in the discovery cohort (GSE65682). (A) All patients, (B) training set, and (C) test set. Plus signs indicate censored observations. Log‐rank *p*‐values are shown. (D–F) Time‐dependent ROC curves for mortality prediction at 1, 2, and 3 weeks. (D) All patients, (E) training set, and (F) test set from GSE65682. AUC values are displayed for each time point. (G) Kaplan–Meier survival curve for external validation cohort (CNP0004962). High‐risk (red) versus low‐risk (blue) groups. Log‐rank *p* = 0.026. (H) Time‐dependent ROC curves for external validation cohort (CNP0004962). AUC values for 1‐, 2‐, and 3‐week mortality prediction. Time‐dependent ROC curves and AUCs in panels D–F and H were estimated using time‐dependent ROC analysis for censored survival data as described in Section 4.

Integrative analysis of single‐cell (GSE167363) [32] and bulk whole‐blood transcriptomic datasets (GSE65682) [33] identified 307 transcripts consistently dysregulated in circulating T cells during sepsis (Figure 5D). From these, Cox screening followed by LASSO Cox regression with 10‐fold cross‐validation yielded a 15‐gene prognostic signature (Figure 5E,F): *TESPA1, SLC11A1, IL1R2, PRKCA, PPM1K, RNF10, MARCO, COX7B, CD96, WHAMM, S1PR1, IER5, COMMD6, LTB, and NUCKS1*. These genes represent coordinated axes of T‐cell dysfunction in sepsis, including dysregulated inflammatory/innate signaling *(IL1R2, LTB, PRKCA, MARCO, SLC11A1, RNF10*, and *COMMD6*), mitochondrial and bioenergetic stress programs (*COX7B, PPM1K, IER5, WHAMM, and NUCKS1*), and impaired trafficking and activation signaling (*S1PR1, TESPA1*, and *CD96*). For each patient, a gene‐derived risk score was calculated as a weighted linear combination of these 15 genes. This score separated high‐risk and low‐risk groups in the discovery cohort (GSE65682), with significantly different 28‐day survival (log‐rank *p* < 0.001; Figure 6A–C) and time‐dependent area under the curve (AUC) values of 0.76–0.85 at 1–3 weeks (Figure 6D–F). The signature demonstrated stable discrimination in external validation (CNP0004962; Figure 6G,H) [34].

### 2.9 Gene‐Clinical Nomogram Enables Accurate Individualized Survival Prediction at ICU Admission

**FIGURE 7:**
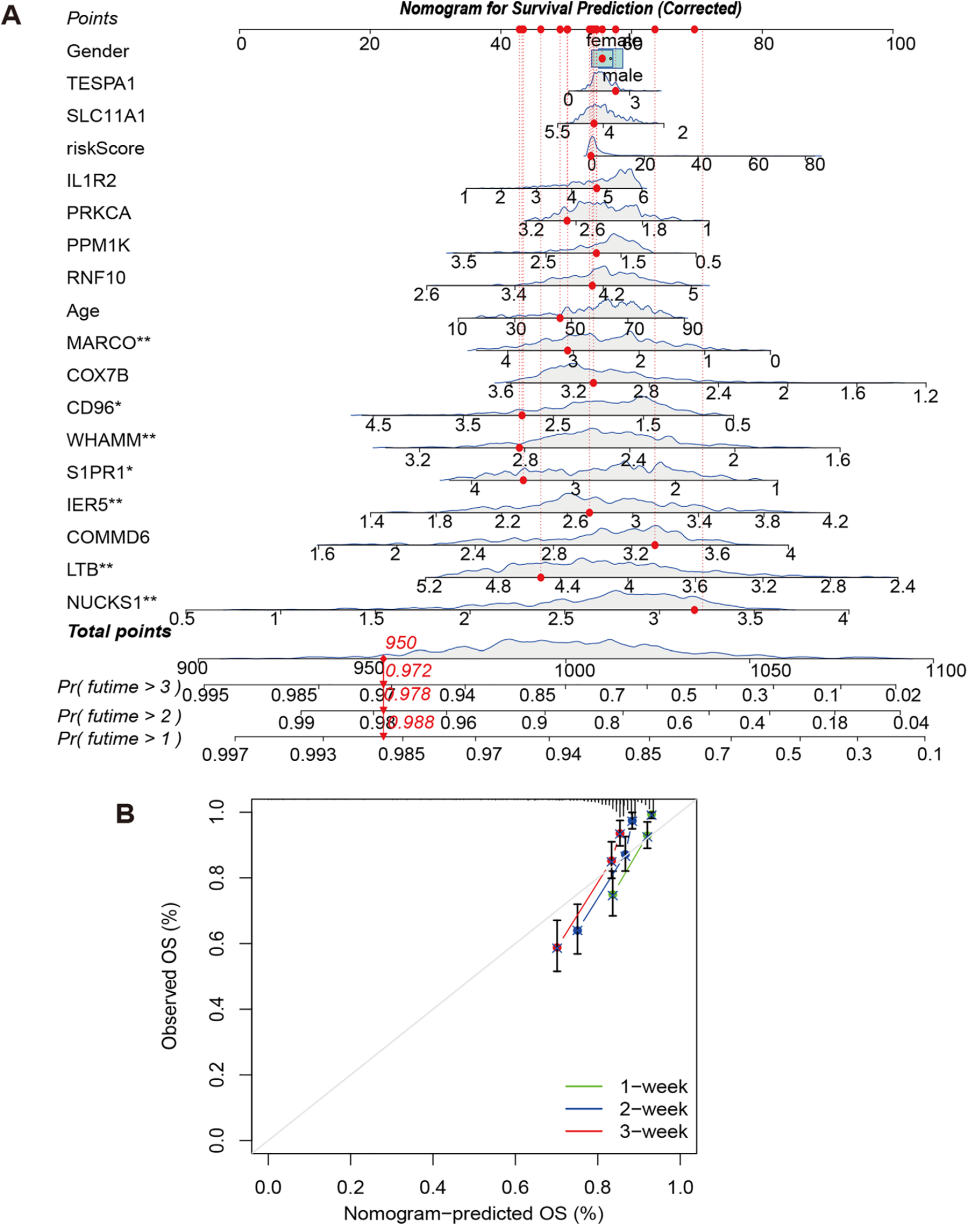
Nomogram for individualized survival prediction integrating the 15‐gene signature and clinical variables. (A) Nomogram for predicting 1‐, 2‐, and 3‐week survival probabilities. The nomogram integrates the 15‐gene risk score (*TESPA1, SLC11A1, IL1R2, PRKCA, PPM1K, RNF10, MARCO, COX7B, CD96, WHAMM, S1PR1, IER5, COMMD6, LTB, NUCKS1*), age, and gender. To calculate locate values on each axis, sum points, and read predicted probabilities from the bottom axes. Asterisks indicate prognostic significance (*p < 0.05, **p < 0.01). Example calculation shown with dashed lines. The nomogram and *p* values for each predictor were derived from a multivariable Cox proportional hazards model. (B) Calibration curves for 1‐, 2‐, and 3‐week predictions. The diagonal dashed line represents perfect calibration. Points near the diagonal indicate good agreement between predicted and observed survival.

To facilitate bedside application, we developed a nomogram integrating the 15‐gene signature with key clinical variables (Figure 7A). The gene‐derived risk score contributed the majority of points in the nomogram, while age and sex provided additional smaller contributions. The nomogram enables clinicians to estimate an individual patient's 1‐, 2‐, and 3‐week survival probabilities at admission. Calibration curves indicated excellent agreement between the nomogram's predictions and actual observed outcomes in the primary cohort (Figure 7B). The nomogram also calibrated well when tested on an external validation cohort, supporting its potential utility for bedside risk stratification (Figure S3).

## 3 Discussion

This study identified immune–inflammatory endotypes that were associated with markedly different survival patterns and with different survival rates among patients who did or did not receive corticosteroids. In patients presenting with lymphopenia and low systemic inflammation, those who received corticosteroids had higher observed survival than those who did not (CD4⁺‐depleted subgroup: 84.4% vs. 75.6%, *p* < 0.001), whereas in hyperinflammatory endotypes, no such difference was apparent regardless of lymphocyte status. Across endotypes, 28‐day survival ranged from 55% to 58% in patients with high inflammation and lymphopenia to 82%–87% in patients with low inflammation and preserved lymphocyte counts. These findings represent associations within an observational cohort and cannot establish causal treatment effects or define steroid‐responsive subgroups. These observations are consistent with evolving concepts that sepsis outcomes are largely determined by a dysregulated host response rather than infection burden alone, underscoring the need for endotype‐guided immunomodulatory strategies in clinical practice [35].

Our findings provide a potential explanation for seemingly contradictory trial results. The ADRENAL trial found no corticosteroid benefit in unselected patients [13], whereas APROCCHSS demonstrated mortality reduction with hydrocortisone plus fludrocortisone [14]. We identified lymphocyte thresholds (374, 340, and 157 cells/µL for total T, CD4⁺, and CD8⁺ cells) where mortality risk increased sharply. These thresholds align with previous observations linking lymphopenia to adverse outcomes [19, 20] and provide specific cut‐points that may facilitate patient stratification. The threshold pattern resembles findings showing that IL‐7 primarily benefits patients with profound lymphopenia [16], consistent with the concept that immune‐directed interventions may demonstrate differential efficacy at specific immune cell concentrations. However, direct comparisons remain limited by differences in the cell populations measured and study designs.

To link these bedside phenotypes to cell‐intrinsic biology, we integrated publicly available single‐cell and bulk blood transcriptomic datasets and derived a 15‐gene T‐cell dysfunction signature that captures qualitative failure states not visible from absolute lymphopenia counts alone. The signature spans coordinated pathophysiological axes. *Immune dysregulation/inflammatory signaling*: *IL1R2* (decoy receptor dampening *IL‐1* responses), *LTB* (lymphotoxin‐β), *PRKCA* and *MARCO* (pattern recognition on activated myeloid cells), and *SLC11A1*, *RNF10*, and *COMMD6* (NF‐κB modulation) indicate perturbed inflammatory control and impaired effector coordination characteristic of sepsis immunoparalysis [36, 37]. *Metabolic/mitochondrial stress*: *COX7B* (respiratory chain), *PPM1K* (mitochondrial phosphatase), and *IER5*/*WHAMM*/*NUCKS1* reflect bioenergetic strain and stress‐adaptation programs; septic T cells shift from oxidative phosphorylation to aerobic glycolysis with compromised ATP supply for proliferation and cytotoxic function [29, 38]. *Trafficking/TCR signaling and functional exhaustion*: *S1PR1* (lymphocyte egress) and *TESPA1* (TCR‐proximal adaptor) capture impaired mobilization and signal transduction that limit antimicrobial competence despite preserved numbers [39, 40]. Therefore, high signature scores index a coordinated collapse of inflammatory control, bioenergetics, and effector capacity within residual T cells rather than simply low lymphopenia counts. Consistent with recent single‐cell studies showing multidimensional disruption of T‐cell fitness in sepsis [28, 29], this signature retained prognostic value across external cohorts (AUC 0.76–0.85). From a practical standpoint, core endotype assignment in this study requires only lymphocyte subset counts and routine inflammatory markers (e.g., CRP and IL‐6), which are already widely available in critical care settings. This accessibility distinguishes our approach from biomarker strategies that depend on specialized molecular platforms. The 15‐gene signature provides mechanistic context and individualized prognostic information but would require transcriptomic infrastructure that is not yet standard in most ICUs, positioning it as an optional refinement rather than a prerequisite for bedside classification.

Several important limitations warrant acknowledgment. The retrospective design precludes causal inference; corticosteroid use was determined by treating clinicians, potentially confounding by indication. Although we adjusted for measured confounders including illness severity, residual confounding from unmeasured factors cannot be excluded. Single‐center derivation limits generalizability. The exclusion of COVID‐19 patients, while necessary to maintain immunopathological homogeneity, restricts direct applicability to SARS‐CoV‐2‐associated sepsis population that may exhibit distinct lymphocyte dynamics, warranting separate investigation. The thresholds were derived using quartile‐based optimization and require validation in independent prospective cohorts; alternative analytical methods might yield different cut‐points. A single time‐point assessment (within 24 h of admission) cannot capture the temporal evolution of immune dysfunction. Our analysis focused on corticosteroids; findings may not apply to other immunomodulatory agents with different mechanisms. These findings generate hypotheses that require testing in randomized controlled trials to establish causality and determine whether phenotype‐guided treatment allocation improves outcomes compared with standard care.

In summary, routine lymphocyte subset counts and inflammatory markers were sufficient to classify sepsis patients into immune–inflammatory endotypes with distinct mortality risk. We also observed differences in survival between corticosteroid‐treated and untreated patients within certain endotypes, but these associations are exploratory and cannot be interpreted as evidence of treatment effect. These findings motivate prospective, endotype‐enriched interventional studies to test whether biology‐based patient stratification can inform immunomodulatory therapy in sepsis.

## 4 Methods

### 4.1 Study Design and Patient Selection

This retrospective cohort study was conducted at Zhongnan Hospital, Wuhan University, following Institutional Review Board approval (Approval number: 2025214K). We included adult patients (≥ 18 years) with sepsis according to Sepsis‐3 criteria [41] who underwent lymphocyte subset analysis within 24 h of ICU admission (January 2020–December 2024).

Accordingly, exclusion criteria were designed to ensure immune assessment validity: hematologic malignancies, HIV infection, primary immunodeficiency, recent immunosuppressive therapy (> 1 mg/kg corticosteroids or biologics within 30 days), pregnancy, confirmed COVID‐19 (distinct viral sepsis phenotype characterized by virus‐driven lymphocyte depletion and T‐cell dysfunction), do‐not‐resuscitate orders, ICU stay < 24 h, or missing critical data. After applying these criteria, 714 patients were included.

### 4.2 Lymphocyte Subset Assessment

Peripheral blood was collected in EDTA tubes and processed within 4 h. Lymphocyte subsets were quantified in the hospital clinical laboratory using a standardized diagnostic TBNK panel (BD Multitest 6‐Color TBNK Reagent with BD Trucount tubes; BD Biosciences) on a BD FACSCanto II flow cytometer. The fixed panel includes pre‐titered, fluorochrome‐conjugated monoclonal antibodies against CD3 (FITC, clone SK7), CD16 (PE, clone B73.1), CD56 (PE, clone NCAM16.2), CD45 (PerCP‐Cy5.5, clone 2D1 [HLe‐1]), CD4 (PE‐Cy7, clone SK3), CD19 (APC, clone SJ25C1), and CD8 (APC‐Cy7, clone SK1). CD3⁺ T cells, CD4⁺ T cells, CD8⁺ T cells, CD19⁺ B cells, and CD16⁺/CD56⁺ NK cells were enumerated, and absolute counts (cells/µL) were obtained using BD Trucount according to the manufacturer's instructions. Cytometer performance was verified by daily calibration and participation in external proficiency testing.

### 4.3 Inflammatory Endotype Classification and Data Management

We derived a composite inflammatory index using PCA on log_10_‐transformed concentrations of PCT, CRP, and IL‐6. The first principal component, which explained 73.2% of the total variance, was selected to represent systemic inflammatory activity and dichotomized at its median to classify patients into high‐ and low‐inflammation groups. Immune status was independently defined by the median total lymphocyte count, which served as a quantitative surrogate of immune competence. By integrating these two dimensions such as systemic inflammation and immune cellularity, we delineated four distinct immune‐inflammatory endotypes. Subsequent analyses evaluated their prognostic relevance and differential response to corticosteroid therapy. Missing data were minimal (< 10% for all key variables). We addressed missing values using multiple imputation by chained equations (MICE) [42] with predictive mean matching. Five imputed datasets were generated and analyzed in parallel, and results were pooled using Rubin's rules to obtain final estimates.

### 4.4 Confounding Adjustment for Corticosteroid Analysis

Because corticosteroid use was not randomized, we addressed confounding by indication using PSM. Propensity scores were modeled to predict steroid receipt from baseline covariates: age, sex, severity scores (APACHE II, SOFA), organ support at admission, infection source and pathogen, admission immune–inflammatory endotype, and treating team (proxy for physician preference). One‐to‐one nearest‐neighbor matching (0.2 SD caliper) achieved covariate balance (standardized mean differences < 0.1). Cox regression with robust standard errors estimated associations with 28‐day mortality in matched cohorts. Sensitivity analyses included inverse probability of treatment weighting (IPTW) and *E*‐value calculation for unmeasured confounding; results are interpreted as adjusted associations, not causal effects.

### 4.5 Assessment of Lymphocyte‐Mortality Associations and Threshold Identification

To characterize the relationship between lymphocyte counts and mortality risk, we first employed locally weighted scatterplot smoothing (LOWESS) curves (smoothing span = 0.75) to visualize the overall risk trend without assuming linearity. We then formally tested for nonlinearity by fitting RCS functions with knots at the fifth, 35th, 65th, and 95th percentiles of lymphocyte counts [43]. Separate logistic regression models with spline‐transformed predictors were used to evaluate their associations of total T‐cell count, CD4⁺ T‐cell count, and CD8⁺ T‐cell count with 28‐day mortality. Optimal lymphocyte subset cutoff values were identified by evaluating risk inflection points using a quartile‐based risk‐optimization approach (testing 25th‐, 50th‐, and 75th‐percentile cut‐points) to maximize the difference in mortality risk between patient groups. The optimal cutoffs were validated by calculating mortality odds ratios and performing chi‐square tests comparing outcomes in patients above versus below each threshold.

### 4.6 scRNA‐seq Analysis

We analyzed publicly available scRNA‐seq data generated from PBMCs collected at sepsis recognition (0 h) from five adult ICU patients with clinically diagnosed sepsis (GEO: GSE167363; sample identifiers are provided in Table S1). Raw UMI count matrices were processed in R (v4.3.0) using Seurat [44]. Cells were retained if nFeature_RNA > 200 and < 5500, nCount_RNA between 1000 and 35,000, and mitochondrial transcript percentage < 10%. After quality control, data were log‐normalized, integrated across patients using FindIntegrationAnchors, reduced by PCA, clustered using FindNeighbors and FindClusters, and visualized via UMAP/t‐SNE. Cell populations were annotated using canonical lineage markers together with SingleR‐based label transfer, identifying major circulating immune populations (including T cells, B cells, monocyte/macrophage‐lineage cells, NK cells, neutrophils, and platelets). T cells (*CD3D*⁺) were further subclustered into CD4⁺ and CD8⁺ populations based on established markers (e.g., *CD4*, *CCR7*, IL7R for CD4⁺ cells; *CD8A*, *CD8B*, *GZMH* for CD8⁺ cells). For each T‐cell subcluster, marker genes were identified using Seurat's FindAllMarkers (Wilcoxon rank‐sum test) with log fold‐change > 0.25, minimum detection fraction > 0.25, and Benjamini–Hochberg adjusted *p* < 0.05. These marker sets were treated as transcriptional features of circulating T‐cell dysfunction in sepsis.

### 4.7 Bulk Transcriptomics and Prognostic Modeling

We next analyzed bulk whole‐blood transcriptomic profiles with linked 28‐day survival from two independent ICU sepsis cohorts: a public dataset (GSE65682) and an institutional cohort (CNP0004962, *n* = 134; baseline characteristics in Table S2). Expression matrices underwent standard preprocessing, including background correction, sample‐level quality control, removal of extremely low‐abundance transcripts, cross‐sample normalization, and batch‐effect adjustment.

To derive a prognostic immune dysfunction signature, we used a multi‐step procedure. First, within each cohort, we identified genes that were significantly dysregulated in sepsis whole blood (adjusted *p* < 0.05). We then intersected these sepsis‐dysregulated genes with the T‐cell transcriptional programs defined by the scRNA‐seq analysis (GSE167363), yielding a biologically focused candidate pool representing circulating T‐cell dysfunction signals. Second, for each gene in this candidate pool, we performed univariate Cox proportional hazards regression using 28‐day mortality as the endpoint to retain genes individually associated with outcome (*p* < 0.05). Third, we entered the retained genes into a penalized Cox model using LASSO [45] with 10‐fold cross‐validation, which selected the minimal set of jointly informative predictors and estimated their coefficients. The genes remaining at the optimal penalty parameter constituted the final 15‐gene prognostic signature.

For each patient, we calculated an individual risk score as a weighted linear combination of the normalized expression levels of these 15 genes, with the LASSO coefficients providing the weights. Model performance was assessed using time‐dependent ROC curves and calibration analyses across clinically distinct cohorts. Full analytic details are provided in the Supporting Information Methods.

### 4.8 Clinical Decision Support Tool

We constructed a nomogram [46] to integrate the molecular risk signature with clinical variables for bedside use. The nomogram provided individualized 1‐, 2‐, and 3‐week survival probabilities based on a patient's gene signature risk score and demographic factors (age and sex). Calibration curves were generated to compare the nomogram‐predicted survival with observed outcomes at each time point, assessing the model's clinical reliability.

### 4.9 Statistical Analysis

Continuous variables are summarized as medians with IQR, and categorical variables as counts and percentages. Group comparisons utilized the Mann–Whitney *U* test for continuous data or the chi‐square test for categorical data. Associations with 28‐day mortality were evaluated using Cox proportional hazards regression, reporting HR with 95% CI. All tests were two‐tailed, with *p* < 0.05 considered statistically significant. Analyses were performed using R version 4.2.1.

## Author Contributions

Zhongyi Sun and Li Li conceived the project. Wenkang Gao and Li Li collected the data. Zhongyi Sun and Wenkang Gao performed the data analysis. Zhongyi Sun wrote and edited the manuscript. Han Gao, Liangyu Guo, and Zhiyong Peng supervised the study and revised the manuscript. All authors have read and approved the final manuscript.

## Funding

This work was supported by the Key R&D Special Project of the Department of Science and Technology of Hubei Province (No. 2023BCB015) and the Noncommunicable Chronic Diseases‐National Science and Technology Major Project (No. 2024ZD0522705).

## Ethics Statement

This study was approved by the Institutional Review Board of Zhongnan Hospital, Wuhan University (Approval number: 2025214K). Written informed consent was waived due to the retrospective nature of the study.

## Conflicts of Interest

The authors declare no conflicts of interest.

## Supporting information




**Table S1**: Details of five Sc‐RNA samples.
**Figure S1**: Quality control and cell type identification from single‐cell RNA sequencing of sepsis patients. (A) Quality control metrics for five sepsis patient samples. Violin plots show nFeature_RNA (genes per cell), nCount_RNA (RNA counts per cell), and percent.mt (mitochondrial RNA percentage) across samples. (B) Correlation between RNA counts and quality metrics. Scatter plots show relationships between nCount_RNA and percent.mt (left, r = −0.22) or nFeature_RNA (right, r = 0.85). Each dot represents a cell, colored by sample. Correlation coefficients (r) were calculated as described in the Methods; no formal hypothesis testing was performed in this figure. (C) UMAP projection of integrated cells colored by cluster (0‐9). (D) UMAP projection colored by cell type. Six cell types identified using SingleR: T cells, B cells, NK cells, monocytes, neutrophils, and platelets. (E)Dot plot of marker gene expression across cell types. Dot size indicates percentage of cells expressing each gene; color intensity represents average expression level. (F) Feature plots showing marker gene expression on UMAP. Representative markers include *IL7R* (T cells), *S100A4* (monocytes), *IGLC2* (B cells), *GNLY* (NK cells), *SERPINB2* (neutrophils), *CCL3* (chemokine), *CD79A* (B cells), *PPBP* (platelets), *LCN2* (neutrophils), and *HBB* (hemoglobin).
**Figure S2**: Immune cell composition and T‐cell subset marker expression. (A) Proportion of six immune cell types across five sepsis patient samples. Cell types are color‐coded: T cells, B cells, NK cells, monocytes, neutrophils, and platelets. Data are descriptive summaries of cell‐type proportions; no formal hypothesis testing was performed. (B) Dot plot of CD4⁺ and CD8⁺ T‐cell marker gene expression. Dot size indicates percentage of cells expressing each gene; color intensity represents average expression level. CD4⁺ markers: *CD4, SELL, CCR7, ITK, IL7R*. CD8⁺ markers: *CD8A, CD8B, CD27, CRTAM, GZMH*. (C) t‐SNE feature plots showing marker gene expression across T‐cell subclusters (0‐9). Color intensity represents expression level. Top row: CD4⁺ markers (*CD4, SELL, CCR7, ITK, IL7R*). Bottom row: CD8⁺ markers (*CD8A, CD8B, CD27, CRTAM, GZMH*).
**Table S2**: Baseline characteristics of 134 sepsis patients by 28‐day survival status
Figure S3. Clinical nomogram for precision medicine application in CNP0004962 cohort. (A) Nomogram integrating gene signature with demographic variables for individualized risk assessment. The nomogram and coefficients were derived from a multivariable Cox proportional hazards model. (B) Calibration curves demonstrating agreement between predicted and observed outcomes across multiple timepoints. Calibration was assessed as described in the Methods.

## Data Availability

De‐identified datasets used in this study are available from the corresponding author upon reasonable request and completion of appropriate institutional data‐sharing agreements. The raw RNA‐sequencing data have been deposited in the China National GeneBank Sequence Archive (CNSA) at CNGBdb under the project accession number CNP0004962 (https://db.cngb.org/cnsa/project/CNP0004962).
