# Supplementary material for: Sepsis Endotypes Defined by Lymphocyte Thresholds and Inflammation Inform Precision Immunomodulation
Source: MedComm (2020). 2026 Jan 4;7(1):e70561. doi: 10.1002/mco2.70561 (PMC12765407; doi:10.1002/mco2.70561)
Supplement: Supplementary file 1 — Table S1: Details of five Sc‐RNA samples. Figure S1: Quality control and cell type identification from single‐cell RNA sequencing of sepsis patients. (A) Quality control metrics for five sepsis patient samples. Violin plots show nFeature_RNA (genes per cell), nCount_RNA (RNA counts per cell), and percent.mt (mitochondrial RNA percentage) across samples. (B) Correlation between RNA counts and quality metrics. Scatter plots show relationships between nCount_RNA and percent.mt (left, r = −0.22) or nFeature_RNA (right, r = 0.85). Each dot represents a cell, colored by sample. Correlation coefficients (r) were calculated as described in the Methods; no formal hypothesis testing was performed in this figure. (C) UMAP projection of integrated cells colored by cluster (0‐9). (D) UMAP projection colored by cell type. Six cell types identified using SingleR: T cells, B cells, NK cells, monocytes, neutrophils, and platelets. (E)Dot plot of marker gene expression across cell types. Dot size indicates percentage of cells expressing each gene; color intensity represents average expression level. (F) Feature plots showing marker gene expression on UMAP. Representative markers include IL7R (T cells), S100A4 (monocytes), IGLC2 (B cells), GNLY (NK cells), SERPINB2 (neutrophils), CCL3 (chemokine), CD79A (B cells), PPBP (platelets), LCN2 (neutrophils), and HBB (hemoglobin). Figure S2: Immune cell composition and T‐cell subset marker expression. (A) Proportion of six immune cell types across five sepsis patient samples. Cell types are color‐coded: T cells, B cells, NK cells, monocytes, neutrophils, and platelets. Data are descriptive summaries of cell‐type proportions; no formal hypothesis testing was performed. (B) Dot plot of CD4⁺ and CD8⁺ T‐cell marker gene expression. Dot size indicates percentage of cells expressing each gene; color intensity represents average expression level. CD4⁺ markers: CD4, SELL, CCR7, ITK, IL7R. CD8⁺ markers: CD8A, CD8B, CD27, CRTAM, GZMH. (C) t‐SNE feature [file MCO2-7-e70561-s001.docx]

Sepsis Endotypes Defined by Lymphocyte Thresholds and Inflammation Inform Precision Immunomodulation
Zhongyi Sun1#, Li Li1#, Wenkang Gao1, Han Gao3*, Liangyu Guo2* Zhiyong Peng1*
1. Department of Critical Care Medicine, Zhongnan Hospital of Wuhan University, Wuhan, Hubei Province 430071, China
Department of Orthopedics, Renmin Hospital of Wuhan University, Wuhan, 430060, China.
3. Department of Pulmonary Medicine, Zhongnan Hospital of Wuhan University, Wuhan, Hubei Province 430071, China.

#Zhongyi Sun, Li Li contributed equally to this work and shared first authorship.
* Corresponding authors:
Han Gao 
mail: 2022103030001@whu.edu.cn
Liangyu Guo
E-mail: wh-synth@tsingke.com.cn
Zhiyong Peng
E-mail: Pengzy5@hotmail.com


Supplement Methods
Single-cell RNA sequencing data acquisition and preprocessing
Single-cell RNA sequencing (scRNA-seq) data were obtained from the Gene Expression Omnibus (GEO; accession GSE167363). For this analysis, we included five peripheral blood mononuclear cell (PBMC) samples collected from five adult ICU patients at the time sepsis was clinically recognized (0 h). Sample identifiers and 28-day survival status are provided in Supplementary Table S1.
Raw UMI count matrices were imported into R (v4.3.0) and converted into Seurat objects using CreateSeuratObject. To exclude low-quality or dying cells, we applied per-cell inclusion thresholds of nFeature_RNA >200 and <5500, nCount_RNA between 1,000 and 35,000, and mitochondrial transcript percentage (percent.mt) <10%. Cells failing these criteria were removed prior to downstream analysis.
Distributions of detected genes per cell, total UMI counts per cell, and mitochondrial transcript percentages for all five sepsis samples are shown in Supplementary Figure S1A-B.

Normalization, integration, dimensionality reduction, and clustering
Following quality control, expression values were log-normalized using Seurat’s NormalizeData. We identified 2,000 highly variable genes (HVGs) using SelectIntegrationFeatures and integrated the five sepsis-derived PBMC samples using FindIntegrationAnchors to mitigate inter-patient batch effects. The integrated expression matrix was then scaled and subjected to principal component analysis (PCA). Shared nearest-neighbor graphs were constructed using FindNeighbors, and unsupervised clustering was performed using FindClusters.
Low-dimensional embeddings were generated using Uniform Manifold Approximation and Projection (UMAP) and t-distributed Stochastic Neighbor Embedding (t-SNE) to visualize the structure of circulating immune cell populations across the five sepsis patients. The integrated UMAP and t-SNE projections, demonstrating separation of major immune populations, are presented in Supplementary Figure S1C-D.

Cell type annotation
Cluster identities were assigned using two complementary approaches: (i) canonical immune lineage markers and (ii) automated label transfer with SingleR. We identified major circulating immune compartments, including: T cells (CD3D⁺), B cells (MS4A1/CD19⁺), monocyte/macrophage-lineage cells (e.g., LST1, S100A8/A9, FCGR3A), NK cells (GNLY, NKG7), neutrophils, and platelets (PF4, PPBP). Consistent with this approach, we identified six major immune cell classes: T cells, B cells, macrophage/monocyte-lineage cells, NK cells, neutrophils, and platelets (Supplementary Figure S1C-F). Lineage-defining marker enrichment patterns across these clusters are summarized in Supplementary Figure S2A. We then isolated T cells and performed subclustering. CD4⁺-enriched subclusters were characterized by expression of CD4, CCR7, IL7R, SELL, and ITK, consistent with helper/memory phenotypes. CD8⁺-enriched subclusters were characterized by CD8A, CD8B, GZMH, CD27, and CRTAM, consistent with cytotoxic/activated phenotypes. Only sepsis patient samples were analyzed; no healthy donor samples were included. Thus, the identified states represent within-sepsis T-cell programs rather than case-control contrasts. T-cell subclustering and the separation of CD4⁺ versus CD8⁺ populations are illustrated in Supplementary Figure S2B, and representative marker expression profiles for these T-cell subclusters are shown in Supplementary Figure S2C.

T-cell subcluster marker identification
Within the annotated T-cell subclusters, we identified subcluster-enriched marker genes using Seurat’s FindAllMarkers with the Wilcoxon rank-sum test. Marker genes were defined as transcripts selectively overexpressed in a given T-cell subcluster relative to other immune compartments and to other T-cell states in the same dataset. Selection thresholds were: log fold-change >0.25, minimum detection fraction (min.pct) >0.25, and Benjamini-Hochberg adjusted P<0.05.
These subcluster-enriched gene sets were interpreted as transcriptional features of circulating T-cell dysfunction in sepsis, spanning dysregulated inflammatory signaling, bioenergetic and mitochondrial stress, altered trafficking and egress, and impaired effector function.

Bulk whole-blood transcriptomic cohorts and preprocessing
To relate circulating T-cell transcriptional programs to clinical outcome, we analyzed two independent ICU sepsis whole-blood transcriptomic cohorts with matched 28-day survival data: a publicly available cohort (GSE65682) and an institutional cohort (CNP0004962, n=134). Baseline clinical characteristics of CNP0004962, including survival stratification, are summarized in Supplementary Table S2.
For each cohort, raw or normalized expression matrices underwent standard preprocessing: background correction, sample-level quality control to exclude outlier samples, removal of extremely low-abundance genes, between-sample normalization (log-transformed expression), and batch-effect adjustment using empirical Bayes-style correction to harmonize technical variation across samples.

Feature selection, survival modeling, and risk score construction
The prognostic signature was constructed in sequential steps.

Biology-guided candidate gene pool.
We first identified genes that were significantly dysregulated in sepsis whole blood (differential expression analysis with multiple-testing correction, adjusted P<0.05). We then intersected these differentially expressed genes with T-cell transcriptional programs defined from the scRNA-seq analysis of septic PBMCs (GSE167363). This intersection yielded a candidate pool of transcripts that (i) map to T-cell dysfunction states observed at single-cell resolution and (ii) are detectably perturbed at the systemic whole-blood level.

Prognostic screening.
For each gene in this candidate pool, we performed univariate Cox proportional hazards regression using 28-day mortality as the endpoint. Genes with prognostic association at P<0.05 were retained for multivariable modeling.

Penalized multivariable model (LASSO-Cox).
The retained prognostic genes were then entered into a penalized Cox proportional hazards model using least absolute shrinkage and selection operator (LASSO) with 10-fold cross-validation. Cross-validation was used to identify the optimal regularization parameter (λ) that balanced model complexity and predictive performance while limiting overfitting. Genes with non-zero coefficients at the optimal λ defined the final 15-gene prognostic signature.

Patient-level risk score and validation.
For each patient, we calculated an individualized risk score as a weighted linear combination of the normalized expression levels of the 15 signature genes, where the LASSO-derived coefficients served as weights. We then evaluated discrimination using time-dependent ROC curves at clinically relevant time points and assessed calibration by comparing predicted versus observed 28-day mortality. To illustrate clinical interpretability, we constructed a nomogram in the CNP0004962 cohort that integrates the gene-derived risk score with demographic and baseline clinical variables (Supplementary Figure S3A). Calibration performance is shown in Supplementary Figure S3B.

Supplement Results
The peripheral blood cell composition in sepsis patients
This study analyzed the heterogeneity of sepsis using the GSE167363 scRNA-seq dataset, focusing on 5 septic patients in the Intensive Care Unit (ICU) at 0 hours from sepsis recognition, as detailed in Table S1. Integrated analysis was conducted for each sample to remove batch differences and low-quality cells. Subsequently, PCA dimension reduction was applied to the integrated data, retaining the top 10 dimensions of the cells. Figure S1A presents a comparison of quality control information among different samples, while Figure S1B shows the results regarding the number of genes, RNA counts, and the correlation between mitochondrial RNA. Through “singleR” packge, we identified six cell types, including T cells (clusters 0, 9), B cells (clusters 2, 6), platelets (cluster 7), macrophages (clusters 1, 4, 5), NK cells (cluster 3), and neutrophils (cluster 8) (Figure S1C, D). Macrophages, T cells, and B cells were present in multiple subclusters, indicating their significant roles in the occurrence and progression of sepsis. The key marker genes for each cell type are shown in Figure S1E, F and Figure S2A. 

Table S1. Details of five Sc-RNA samples.
Patient ID	Time from sepsis recognition	disease state	Sample source	Sample treatment protocol
GSM5102902	0 hours	non-survived	PBMCs	No growth or treatment performed
GSM5102904	0 hours	survived	PBMCs	No growth or treatment performed
GSM5511351	0 hours	non-survived	PBMCs	No growth or treatment performed
GSM5511353	0 hours	survived	PBMCs	No growth or treatment performed
GSM5511355	0 hours	survived	PBMCs	No growth or treatment performed


FigureS1. Quality control and cell type identification from single-cell RNA sequencing of sepsis patients. (A) Quality control metrics for five sepsis patient samples. Violin plots show nFeature_RNA (genes per cell), nCount_RNA (RNA counts per cell), and percent.mt (mitochondrial RNA percentage) across samples. (B) Correlation between RNA counts and quality metrics. Scatter plots show relationships between nCount_RNA and percent.mt (left, r=−0.22) or nFeature_RNA (right, r=0.85). Each dot represents a cell, colored by sample. Correlation coefficients (r) were calculated as described in the Methods; no formal hypothesis testing was performed in this figure. (C) UMAP projection of integrated cells colored by cluster (0-9). (D) UMAP projection colored by cell type. Six cell types identified using SingleR: T cells, B cells, NK cells, monocytes, neutrophils, and platelets. (E)Dot plot of marker gene expression across cell types. Dot size indicates percentage of cells expressing each gene; color intensity represents average expression level. (F) Feature plots showing marker gene expression on UMAP. Representative markers include IL7R (T cells), S100A4 (monocytes), IGLC2 (B cells), GNLY (NK cells), SERPINB2 (neutrophils), CCL3 (chemokine), CD79A (B cells), PPBP (platelets), LCN2 (neutrophils), and HBB (hemoglobin).

Differential Gene Expression in CD4⁺ and CD8⁺ T-cell Subsets
To characterize the cellular composition of peripheral blood in sepsis patients, we first examined the relative abundance of six major immune cell types across five individual samples (Figure S2A). monocytes constituted the predominant population, followed by T cells with notable inter-patient variability in cellular composition. Sample GSM5511353 exhibited the highest T-cell proportion with correspondingly reduced monocyte frequency, while sample GSM5102902 showed a more balanced distribution with elevated monocyte and neutrophil populations.
Given the prognostic significance of T-cell counts demonstrated in our clinical cohort, we performed higher-resolution analysis of T-cell subsets. Unsupervised reclustering of CD3D⁺ T cells resolved 10 distinct subclusters with differential expression of canonical CD4⁺ and CD8⁺ lineage markers (Figure S2B). CD4⁺ T cells were characterized by expression of CD4, SELL, CCR7, ITK, and IL7R, consistent with helper and central memory phenotypes. In contrast, CD8⁺ T cells demonstrated enriched expression of CD8A, CD8B, CD27, CRTAM, and GZMH, indicative of cytotoxic effector states. (FigureS 2C). 
Figure S2. Immune cell composition and T-cell subset marker expression. (A) Proportion of six immune cell types across five sepsis patient samples. Cell types are color-coded: T cells , B cells, NK cells, monocytes, neutrophils, and platelets. Data are descriptive summaries of cell-type proportions; no formal hypothesis testing was performed. (B) Dot plot of CD4⁺ and CD8⁺ T-cell marker gene expression. Dot size indicates percentage of cells expressing each gene; color intensity represents average expression level. CD4⁺ markers: CD4, SELL, CCR7, ITK, IL7R. CD8⁺ markers: CD8A, CD8B, CD27, CRTAM, GZMH. (C) t-SNE feature plots showing marker gene expression across T-cell subclusters (0-9). Color intensity represents expression level. Top row: CD4⁺ markers (CD4, SELL, CCR7, ITK, IL7R). Bottom row: CD8⁺ markers (CD8A, CD8B, CD27, CRTAM, GZMH).


Baseline characteristics of 134 sepsis patients
Among the 134 patients, non-survivors had a higher prevalence of coronary artery disease (42.1% vs. 13.5%, P < 0.001), heart failure (36.8% vs. 18.7%, P = 0.027), COPD (26.3% vs. 11.5%, P = 0.033), kidney disease (28.9% vs. 13.5%, P = 0.036), and lung infection (26.3% vs. 11.5%, P = 0.033) compared with survivors. Hemoglobin levels were significantly lower in non-survivors (86.0 vs. 107.5 g/L, P < 0.001), whereas age, sex, illness severity scores, inflammatory markers, and coagulation parameters showed no significant differences between the two groups.

Table S2. Baseline characteristics of 134 sepsis patients by 28-day survival status
Variables	Total (n = 134)	Survival  (n = 96)	Non-Survival (n = 38)	P
Demographics				
Age, years	67.0 (57.3, 75.0)	66.5 (57.0, 75.0)	70.50 (58.3, 75.0)	0.744
Male, n (%)	60 (44.8)	42 (43.7)	18 (47.4)	0.704
Severity scores				
APACHE2	20.60 (16.0, 27.9)	19.70 (16.0, 27.0)	23.6 (18.0, 29.3)	0.162
SOFA	8.0 (5.0, 15.0)	7.5 (5.0, 14.0)	8.0 (6.0, 16.0)	0.304
Comorbidities				
Hypertension, n(%)	69 (51.5)	46 (47.9)	23 (60.5)	0.188
Diabetes, n(%)	41 (30.6)	32 (33.3)	9 (23.7)	0.275
CAD, n(%)	29 (21.6)	13 (13.5)	16 (42.1)	<.001
Heart failure, n(%)	32 (23.9)	18 (18.7)	14 (36.8)	0.027
Stroke, n(%)	42 (31.3)	27 (28.1)	15 (39.5)	0.202
COPD, n(%)	21 (15.7)	11 (11.5)	10 (26.3)	0.033
Kidney disease, n(%)	24 (17.9)	13 (13.5)	11 (28.9)	0.036
Lung infection, n(%)	21 (15.7)	11 (11.5)	10 (26.3)	0.033
Organ Function & Metabolic				
WBC, ×10³/µL	14.5 (9.9, 21.7)	14.09 (9.7, 21.0)	15.40 (12.3, 20.9)	0.307
Platelets, ×10³/µL	132.0 (63.3, 201.8)	136.5 (64.8, 209.0)	101.0 (43.5, 193.8)	0.302
Neutrophil , n(%)	84.5 (54.9, 91.6)	86.6 (56.7, 92.1)	82.2 (23.8, 91.0)	0.328
Lymphocyte, n(%)	4.2 (1.7, 8.8)	4.1 (1.6, 9.0)	4.2 (1.7, 8.6)	0.929
Hemoglobin,g/L	102.7 (81.2, 122.8)	107.5 (85.2, 126.5)	86.0 (68.0, 109.3)	<.001
ALT,U/L	29.2 (15.3, 58.7)	28.5 (15.0, 59.9)	35.0 (17.5, 58.2)	0.894
AST,U/L	39.90 (25.3, 80.5)	39.4 (25.8, 81.3)	42.1 (26.0, 72.8)	0.927
TBIL,umol/L	16.8 (11.1, 44.5)	167.0 (11.0, 35.4)	16.1 (11.4, 63.9)	0.627
ALB,g/L	29.0 (26.3, 33.3)	29.1 (26.5, 33.7)	28.9 (24.6, 31.9)	0.366
BUN, mmol/L	12.9 (7.1, 18.7)	13.5 (7.5, 19.2)	12.2 (6.1, 18.2)	0.386
Cr, μmol/L	127.5 (77.9, 206.1)	119.6 (79.8, 206.2)	140.7 (73.0, 196.5)	0.863
UA, μmol/L	299.9 (199.3, 430.5)	336.9 (217.0, 430.4)	249.5 (175.3, 451.5)	0.181
Lactate, mmol/L	2.5 (1.6, 4.5)	2.50 (1.7, 4.5)	2.6 (1.4, 4.8)	0.577
PCT, ng/mL	17.02 (2.4, 48.8)	17.0 (2.3, 50.9)	17.0 (4.1, 42.1)	0.949
Coagulation				
Fibrinogen, g/L	6.3 (4.3, 362.8)	6.7 (4.8, 392.6)	5.3 (4.0, 9.4)	0.130
PT, sec	15.0 (13.3, 17.4)	14.8 (13.0, 17.3)	15.1 (13.7, 17.8)	0.504
D-dimer, ng/mL	2.6 (1.4, 7.2)	2.5 (1.1, 6.6)	3.4 (2.0, 8.1)	0.101
INR	1.3 (1.2, 1.5)	1.3 (1.2, 1.5)	1.2 (1.1, 1.5)	0.592


Figure S3. Clinical nomogram for precision medicine application in CNP0004962 cohort. (A) Nomogram integrating gene signature with demographic variables for individualized risk assessment. The nomogram and coefficients were derived from a multivariable Cox proportional hazards model. (B) Calibration curves demonstrating agreement between predicted and observed outcomes across multiple timepoints. Calibration was assessed as described in the Methods.
